# Racial and Ethnic Differences in Hospice Use and Hospitalizations at End-of-Life Among Medicare Beneficiaries With Dementia

**DOI:** 10.1001/jamanetworkopen.2022.16260

**Published:** 2022-06-09

**Authors:** Pei-Jung Lin, Yingying Zhu, Natalia Olchanski, Joshua T. Cohen, Peter J. Neumann, Jessica D. Faul, Howard M. Fillit, Karen M. Freund

**Affiliations:** 1 Center for the Evaluation of Value and Risk in Health, Institute for Clinical Research and Health Policy Studies, Tufts Medical Center, Boston, Massachusetts; 2 Survey Research Center, Institute for Social Research, University of Michigan, Ann Arbor; 3 Alzheimer’s Drug Discovery Foundation, New York, New York; 4 Center for Health Equity Research, Department of Medicine, Tufts Medical Center, Tufts University School of Medicine, Boston, Massachusetts

## Abstract

**Importance:**

The pool of studies examining ethnic and racial differences in hospice use and end-of-life hospitalizations among patients with dementia is limited and results are conflicting, making it difficult to assess health care needs of underresourced racial and ethnic groups.

**Objective:**

To explore differences in end-of-life utilization of hospice and hospital services among patients with dementia by race and ethnicity.

**Design, Setting, and Participants:**

This cohort study used national survey data from the Health and Retirement Study linked with Medicare and Medicaid claims that reflected a range of socioeconomic, health, and psychosocial characteristics. Eligible participants were Medicare fee-for-service beneficiaries aged 65 years or older diagnosed with dementia who died between 2000 and 2016. Analyses were performed from June to December 2021.

**Exposures:**

Race and ethnicity.

**Main Outcomes and Measures:**

We examined the frequency and costs of hospice care, emergency department (ED) visits, and hospitalizations during the last 180 days of life among Medicare decedents with dementia. We analyzed the proportion of dementia decedents with advance care planning and their end-of-life care preferences.

**Results:**

The cohort sample included 5058 beneficiaries with dementia (mean [SD] age, 85.5 [8.0] years; 3038 women [60.1%]; 809 [16.0%] non-Hispanic Black, 357 [7.1%] Hispanic, and 3892 non-Hispanic White respondents [76.9%]). In adjusted analysis, non-Hispanic Black decedents (odds ratio [OR], 0.65; 95% CI, 0.55-0.78), nursing home residents (OR, 0.81; 95% CI, 0.71-0.93), and survey respondents represented by a proxy (OR, 0.84; 95% CI, 0.71-0.99) were less likely to use hospice, whereas older decedents (age 75-84 vs 65-74 years: OR, 1.39; 95% CI, 1.12-1.72; age ≥85 vs 65-74 years: OR, 1.39; 95% CI, 1.13-1.71), women (OR, 1.19; 95% CI, 1.05-1.35), and decedents with higher education (high school vs less than high school: OR, 1.17; 95% CI, 1.01-1.36; more than high school vs less than high school: OR, 1.32; 95% CI, 1.13-1.54), more severe cognitive impairment (OR, 1.51; 95% CI, 1.02-2.23), and more instrumental activities of daily living limitations (OR, 1.07; 95% CI, 1.01-1.12) were associated with higher hospice enrollment. A higher proportion of Black and Hispanic decedents with dementia used ED (645 of 809 [79.7%] and 274 of 357 [76.8%] vs 2753 of 3892 [70.7%]; *P* < .001) and inpatient services (625 of 809 [77.3%] and 275 of 357 [77.0%] vs 2630 of 3892 [67.5%]; *P* < .001) and incurred roughly 60% higher inpatient expenditures at the end of life compared with White decedents (estimated mean: Black, $23 279; 95% CI, $20 690-$25 868; Hispanic, $23 471; 95% CI, $19 532-$27 410 vs White, $14 609; 95% CI, $13 800-$15 418). A higher proportion of Black and Hispanic than White beneficiaries with dementia who were enrolled in hospice were subsequently admitted to the ED (56 of 309 [18.1%] and 22 of 153 [14.4%] vs 191 of 1967 [9.7%]; *P* < .001) or hospital (48 of 309 [15.5%] and 17 of 153 [11.1%] vs 119 of 1967 [6.0%]; *P* < .001) before death. The proportion of dementia beneficiaries completing advance care planning was lower among Black (146 of 704 [20.7%]) and Hispanic (66 of 308 [21.4%]) beneficiaries compared with White beneficiaries (1871 of 3274 [57.1%]). A higher proportion of Black and Hispanic decedents with dementia had written instructions choosing all care possible to prolong life (30 of 144 [20.8%] and 12 of 65 [18.4%] vs 72 of 1852 [3.9%]), whereas a higher proportion of White decedents preferred to limit care in certain situations (1708 of 1840 [92.8%] vs 114 of 141 [80.9%] and 51 of 64 [79.7%]), withhold treatments (1448 of 1799 [80.5%] vs 87 of 140 [62.1%] and 41 of 62 [66.1%]), and forgo extensive life-prolonging measures (1712 of 1838 [93.1%] vs 120 of 138 [87.0%] and 54 of 65 [83.1%]).

**Conclusions and Relevance:**

The results of this cohort study highlight unique end-of-life care utilization and treatment preferences across racial and ethnic groups among patients with dementia. Medicare should consider alternative payment models to promote culturally competent end-of-life care and reduce low-value interventions and costs among the population with dementia.

## Key Points

**Question:**

How does use of hospice and hospital services at the end of life differ by race and ethnicity among patients with dementia?

**Findings:**

This cohort study found that non-Hispanic Black and Hispanic decedents with dementia used less hospice but more emergency department and inpatient services, and incurred roughly 60% higher Medicare inpatient expenditures at the end of life, compared with non-Hispanic White decedents. The proportion of dementia beneficiaries completing advance care planning was significantly lower among non-Hispanic Black and Hispanic decedents compared with non-Hispanic White decedents.

**Meaning:**

These results highlight the importance of understanding how end-of-life care utilization and treatment preferences among patients with dementia differ across racial and ethnic groups.

## Teaser

This cohort study examines Medicare and Medicaid claims data alongside sociodemographic data from national surveys to differences in the utilization of end-of-life care services by race and ethnicity.

## Introduction

Despite advances in dementia prevention and treatment,^1^ end-of-life care remains critically important for all individuals living with the condition. Hospice, for example, provides comfort care and support for terminal patients and their families. Older adults with dementia are among those who are most likely to benefit from end-of-life hospice care because these individuals often experience multiple distressing symptoms (eg, pain, depression, and delusions) and poor quality of life.^2^ To qualify for the Medicare hospice benefit, a patient must be certified by 2 physicians as terminally ill, defined as having a medical prognosis of 6 months or less.^3^ However, prognostic challenges in dementia, such as its long disease duration and unpredictable trajectories, make certifying eligibility for the Medicare hospice benefit difficult. Although hospice utilization in the Medicare population has increased in recent decades, the service remains underused, with only half of Medicare decedents enrolling in the hospice benefit.^3,4,5^ Individuals with noncancer illness, including those with dementia, and from underserved communities have lower hospice utilization rates.^5,6,7,8^

Some evidence suggests that, among individuals with dementia, non-Hispanic Black individuals experience more aggressive end-of-life care (eg, artificial nutrition, mechanical ventilation, and intensive care unit admissions) compared with non-Hispanic White individuals.^9,10,11^ However, the pool of studies examining racial and ethnic differences in hospice use and end-of-life hospitalizations among patients with dementia is limited and the results conflicting.^9^ For example, one analysis of nursing home residents with advanced dementia in Boston found that Black individuals were more likely than White to receive hospice referrals,^12^ whereas a US national study suggested that hospice use was lower among Black residents.^13^ The type and intensity of care individuals receive at the end of life may differ for those with more advanced dementia,^10^ but claims-based analyses lack sufficient clinical detail to characterize cognitive function. Furthermore, while studies have examined dementia end-of-life care, the analyses often are restricted to nursing home residents.^14,15,16^ These estimates are limited because they omit a substantial proportion of dementia patients living at home or other settings prior to death.^6^ The samples typically lack racial and ethnic diversity, and thus robust data on non-White decedents with dementia are sparse.^9^ For example, prior examinations of hospice use have compared differences between Black and White patients with dementia but lacked data on other racial or ethnic groups.^14,17,18^ The limitations of previous analyses, such as a lack of dementia severity measures and use of unrepresentative samples, make it difficult to assess unique end-of-life care needs for underresourced racial and ethnic groups.

This study examined the frequency and costs of end-of-life hospice care, emergency department (ED) visits, and hospitalizations among Medicare decedents with dementia. Our study extends existing research by analyzing how dementia end-of-life care utilization patterns differ by race and ethnicity. We supplemented claims data with national survey data that included measures of cognitive function and socioeconomic factors known to affect end-of-life care. We also analyzed ethnic and racial differences in patient instructions regarding end-of-life treatment based on survey data. Our sample included community-dwelling patients and nursing home residents, thus making our findings more representative of the general dementia population. We hypothesized that non-Hispanic Black and Hispanic patients with dementia had lower hospice use but more ED and hospital admissions at the end of life compared with non-Hispanic White patients.

## Methods

The Tufts Medical Center/Tufts University Health Sciences institutional review board approved this cohort study via expedited review procedures per 45 CFR 46.110(b)(4); informed consent was not required because the study analyzed existing, secondary data. We followed the Strengthening the Reporting of Observational Studies in Epidemiology (STROBE) reporting guideline.

### Data and Sample

We used data from the 2000-2016 Health and Retirement Study (HRS) linked with Medicare and Medicaid claims. The HRS is a nationally representative biennial survey of US adults aged 50 or older, collecting a range of respondent socioeconomic, health, and psychosocial characteristics.^19^ Each wave of HRS interviews roughly 20 000 respondents, with response rates ranging from 81% to 89%.^20^ The HRS oversamples Black and Hispanic households, making it well suited for our investigation of racial and ethnic disparities. We used HRS survey data linked to respondents’ Medicare fee-for-service part A (ie, inpatient, skilled nursing facility, hospice, and home health), part B (ie, physician visit, outpatient care, and durable medical equipment), and part D (ie, prescription drug) claims, as well as Medicaid analytic extracts and summary files for dually eligible beneficiaries. These health insurance claims included diagnosis codes, medical and prescription drug utilization, date of service, and reimbursement for all services rendered.

Our sample included HRS respondents aged 65 years and older diagnosed with dementia who died between 2000 and 2016 (eFigure in the Supplement). We identified individuals with a claims-based diagnosis of dementia during the study period by using *International Classification of Diseases*, *Ninth* or *Tenth Revisions* diagnosis codes.^21,22^ We required all patients to have continuous enrollment in Medicare fee-for-service for at least 6 months prior to death.

### Outcome Measures

#### Hospice

We used HRS-linked Medicare claims to analyze the proportion of dementia decedents enrolling in hospice during the last 180 days of life and their cumulative hospice days and hospice expenditures paid by Medicare. We also examined late hospice enrollment (within 7 and 3 days before death) as an indicator of poor end-of-life care quality.

#### ED Visits and Hospitalizations

We examined the average number of outpatient ED visits, inpatient ED visits, and hospital stays during the last 180 days of life, as well as Medicare expenditures for these visits. We calculated total expenditures for inpatient ED and hospital admissions combined because those payments cannot be separately identified in Medicare claims.

We identified major causes of hospitalizations based on principal discharge diagnoses, using the Agency for Healthcare Research and Quality (AHRQ) Clinical Classifications Software (CCS) and Clinical Classifications Software Refined (CCSR) tools.^12,23^ Additionally, we examined the proportion of potentially avoidable hospitalizations for ambulatory care sensitive chronic conditions (ie, hypertension, diabetes, asthma, chronic obstructive pulmonary disease, and heart failure) and acute infections (ie, bacterial pneumonia and urinary tract infection), as defined by the AHRQ Prevention Quality Indicators, version 2021.^24^

#### End-of-Life Care Preferences

We examined patient end-of-life care preferences using the HRS exit interview data reported by a proxy within 2 years after the study participant’s death.^25^ The interview asked whether the participant had advance care planning, ie, written instructions specifying the treatment or care they wanted to receive during the final days of life. Of those study participants with advance care planning, the interview asked whether these instructions expressed any desire to: (1) receive all care possible to prolong life; (2) limit care in certain situations; (3) have any treatment withheld; or (4) keep them comfortable and pain free but forego extensive measures to prolong life (choices not mutually exclusive; each categorized as yes or no).

### Statistical Analysis

We developed 2-part models to assess whether Medicare expenditures for hospice, outpatient ED, and inpatient services differed by race and ethnicity, categorized as non-Hispanic White, non-Hispanic Black, and Hispanic. The first part used a logistic regression to estimate the probability of any use; the second part used a generalized linear model (GLM) with log link and γ distribution to estimate mean expenditures among users. Combining results from these 2 model components yielded population expenditure estimates. We standardized all Medicare expenditures to 2016 US$.

Covariates in both logistic regression and GLM included age, sex, education, cognition, functional limitations, comorbidities, Medicare-Medicaid dual eligibility, nursing home status, and proxy respondent. We measured cognition using HRS-imputed Telephone Interview for Cognitive Status (TICS) scores for self-respondents and Informant Questionnaire on Cognitive Decline in the Elderly (IQCODE) scores for those study participants represented by a proxy. We quantified functional status in terms of the number of limitations in activities of daily living (ADL; including getting dressed, walking across the room, bathing, eating, getting in and out of bed, and using the toilet) and instrumental ADL (IADL; including preparing meals, shopping for groceries, using the telephone, taking medication, and managing one’s money). For end-of-life care preferences, we analyzed the proportion of beneficiaries with advance care planning and their specific instructions (as detailed in “Outcome measures”) by race and ethnicity. All analyses were performed from June to December 2021.

## Results

### Sample Characteristics

Our study sample included 5058 beneficiaries with dementia; 3892 beneficiaries (76.9%) were non-Hispanic White, 809 (16.0%) were non-Hispanic Black, and 357 (7.1%) were Hispanic. Overall, the mean (SD) age of beneficiaries was 85.5 (8.0) years and 3038 participants were women (60.1%) (Table 1). Compared with Black and Hispanic decedents, White decedents with dementia were older (mean age [SD]: 85.9 [7.7] years vs Black, 84.0 [8.8] years and Hispanic, 84.0 [8.6] years; *P* < .001), had higher education levels (1234 of 3892 [31.7%] completed more than high school vs 117 of 809 Black decedents [14.5%] and 40 of 357 Hispanic decedents [11.2%]; *P* < .001), better cognitive scores (mean [SD] TICS scores: 15.9 [6.2] vs Black, 12.5 [6.0] and Hispanic, 13.6 [5.3]; mean [SD] IQCODE scores: 4.1 [0.8] vs 3.9 [0.8] and 4.0 [0.8]; *P* < .001), and fewer functional limitations (eg, mean [SD] IADL limitations: 2.1 [1.8] vs Black, 2.2 [1.9] and Hispanic, 2.3 [1.9]; *P* = .006), were more likely to live in a nursing home (1805 of 3666 [49.2%] vs 336 of 766 Black decedents [43.9%] and 127 of 341 Hispanic decedents [37.2%]; *P* < .001) and less likely to be dually eligible for Medicare and Medicaid (741 of 3892 [19.0%] vs 286 of 809 Black decedents [35.4%] and 169 of 357 Hispanic decedents [47.3%]; *P* < .001).

**Table 1.** Sample Characteristics

Characteristic	Respondents, No. (%)				*P* value
	Total (n = 5058)	Non-Hispanic White (n = 3892)	Non-Hispanic Black (n = 809)	Hispanic (n = 357)	
Age, mean (SD), y	85.5 (8.0)	85.9 (7.7)	84.0 (8.8)	84.0 (8.6)	<.001
Age category, y					
65-74	587 (11.6)	381 (9.8)	148 (18.3)	58 (16.3)	<.001
75-84	1667 (33.0)	1256 (32.3)	279 (34.5)	132 (37.0)	
≥85	2804 (55.4)	2255 (57.9)	382 (47.2)	167 (46.8)	
Sex					
Women	3038 (60.1)	2325 (59.7)	509 (62.9)	204 (57.1)	.12
Men	2020 (39.9)	1567 (40.3)	300 (37.1)	153 (42.9)	
Education					
<High school	2124 (42.0)	1322 (34.0)	527 (65.1)	275 (77.0)	<.001
High school	1543 (30.5)	1336 (34.3)	165 (20.4)	42 (11.8)	
>High school	1391 (27.5)	1234 (31.7)	117 (14.5)	40 (11.2)	
Medicare-Medicaid dual eligibility	1196 (23.7)	741 (19.0)	286 (35.4)	169 (47.3)	<.001
Nursing home resident	2268 (47.5)	1805 (49.2)	336 (43.9)	127 (37.2)	<.001
Proxy respondent	2013 (42.4)	1494 (40.9)	350 (46.1)	169 (49.6)	<.001
TICS scores, mean (SD)^a^	15.3 (6.2)	15.9 (6.2)	12.5 (6.0)	13.6 (5.3)	<.001
IQCODE scores, mean (SD)^a^	4.1 (0.8)	4.1 (0.8)	3.9 (0.8)	4.0 (0.8)	<.001
ADL limitations, mean (SD)	2.1 (2.1)	2.1 (2.1)	2.1 (2.1)	2.4 (2.2)	.04
IADL limitations, mean (SD)	2.1 (1.8)	2.1 (1.8)	2.2 (1.9)	2.3 (1.9)	.006
Comorbidities, mean (SD)^b^	3.3 (1.6)	3.3 (1.6)	3.4 (1.5)	3.4 (1.6)	.17

Abbreviations: ADL, activities of daily living; IADL, instrumental activities of daily living; IQCODE, Informant Questionnaire on Cognitive Decline in the Elderly; TICS, Telephone Interview for Cognitive Status.

^a^ TICS scores were available for self-respondents (2702 respondents) and IQCODE scores were available for those represented by a proxy (1763 respondents).

^b^ Comorbidity count ranged from 0 to 8, including high blood pressure, diabetes, cancer, lung disease, heart disease, stroke, psychiatric problems, and arthritis, based on HRS survey data.

### Hospice Care by Race and Ethnicity

Less than half of the decedents with dementia in our sample (2429 of 5058 [48.0%]) used hospice care in their last 180 days of life (Table 2). The proportion was lower among Black and Hispanic compared with White decedents (309 of 809 [38.2%] and 153 of 357 [42.9%] vs 1967 of 3892 [50.5%], *P* < .001). In adjusted analysis, Black decedents (odds ratio [OR]: 0.65; 95% CI, 0.55-0.78), nursing home residents (OR, 0.81; 95% CI, 0.71-0.93), and respondents represented by a proxy were less likely to use hospice (OR, 0.84; 95% CI, 0.71-0.99), whereas older age (75-84 vs 65-74 years: OR, 1.39; 95% CI, 1.12-1.72; ≥85 vs 65-74 years: OR, 1.39; 95% CI, 1.13-1.71), women (OR, 1.19; 95% CI, 1.05-1.35), higher education (high school vs less than high school: OR, 1.17; 95% CI, 1.01-1.36; more than high school vs less than high school: OR, 1.32; 95% CI, 1.13-1.54), more severe cognitive impairment (OR, 1.51; 95% CI, 1.02-2.23), and more IADL limitations (OR, 1.07; 95% CI, 1.01-1.12) were associated with higher hospice enrollment (eTable 1 in the Supplement).

**Table 2.** Unadjusted Hospice Use, Emergency Department Visits, and Hospitalizations in the Last 180 Days of Life and End-of-Life Care Preferences Among Dementia Decedents by Race and Ethnicity

End-of-life service	Respondents, No. (%)				*P* value
	All (N = 5058)	Non-Hispanic White (n = 3892)	Non-Hispanic Black (n = 809)	Hispanic (n = 357)	
Hospice care					
Any hospice enrollment	2429 (48.0)	1967 (50.5)	309 (38.2)	153 (42.9)	<.001
Total hospice days, mean (SD), d^a^	49.9 (61.3)	50.4 (61.8)	50.1 (59.9)	43.5 (57.4)	.41
Hospice enrollment only during the last 7 d of life^a^	735 (30.3)	606 (30.8)	84 (27.2)	45 (29.4)	.42
Hospice enrollment only during the last 3 d of life^a^	334 (13.8)	269 (13.7)	43 (13.9)	22 (14.4)	.97
ED visits					
Any visit after hospice enrollment	269 (11.1)	191 (9.7)	56 (18.1)	22 (14.4)	<.001
Any visit	3672 (72.6)	2753 (70.7)	645 (79.7)	274 (76.8)	<.001
Total No. of outpatient visits, mean (SD)^a^	2.5 (2.1)	2.5 (2.0)	2.8 (2.2)	2.4 (2.1)	.06
Total No. of inpatient visits, mean (SD)^a^	1.8 (1.2)	1.7 (1.1)	2.2 (1.4)	1.9 (1.1)	<.001
Hospitalization					
Any hospitalization after hospice enrollment	184 (7.6)	119 (6.1)	48 (15.5)	17 (11.1)	<.001
Any hospitalization	3530 (69.8)	2630 (67.6)	625 (77.3)	275 (77.0)	<.001
Total No. of hospitalizations, mean (SD)^a^	2.1 (1.4)	2.0 (1.3)	2.4 (1.5)	2.3 (1.5)	<.001
Length of stay, mean (SD), d^a^	8.5 (7.0)	8.0 (6.6)	10.0 (8.5)	9.6 (6.6)	<.001
Advance care planning (4286 respondents)	2083 (48.6)	1871 (57.2)	146 (20.7)	66 (21.4)	NA
All care possible to prolong life (2061 respondents)	114 (5.5)	72 (3.9)	30 (20.8)	12 (18.5)	NA
Limited care (2045 respondents)	1873 (91.6)	1708 (92.8)	114 (80.9)	51 (79.7)	NA
Any treatment withheld (2001 respondents)	1576 (78.8)	1448 (80.5)	87 (62.1)	41 (66.1)	NA
Comfortable and pain free but forego extensive measures to prolong life (2041 respondents)	1886 (92.4)	1712 (93.1)	120 (87.0)	54 (83.1)	NA

Abbreviation: ED, emergency department.

These are unadjusted, descriptive statistics. *P* values were based on χ^2^ tests (categorical variables) and ANOVA tests (continuous variables).

^a^ Among users.

**Figure 1.**
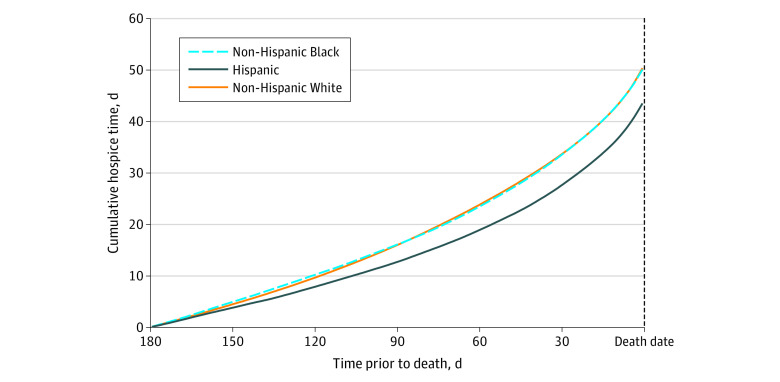
Cumulative Hospice Days in the Last 180 Days of Life Among Dementia Beneficiaries Enrolling in Hospice Care, by Race and Ethnicity

**Figure 2.**
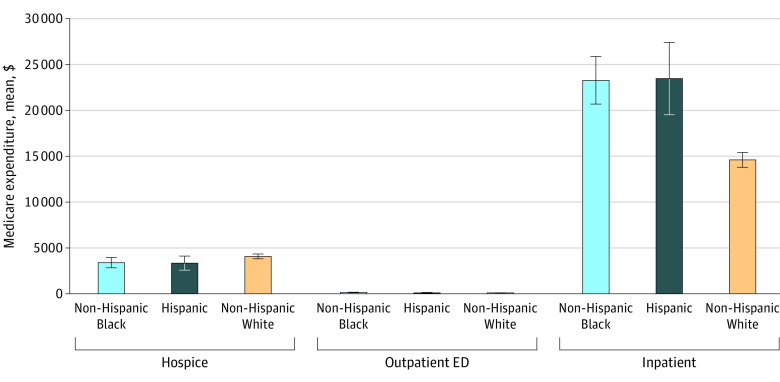
Estimated Mean Medicare Expenditures per Dementia Decedent in the Last 180 Days of Life ED indicates emergency department. Whiskers represent 95% CIs.

Among hospice users, the mean (SD) length of stay was 1.6 (2.0) months, and Hispanic decedents with dementia had the shortest stays (mean [SD] stay: White, 50.4 [61.8] days; Black, 50.1 [59.9] days; Hispanic, 43.5 [57.4] days; *P* = .41) (Figure 1). Late hospice enrollment was common across all racial and ethnic groups; overall, 735 of 2429 beneficiaries with dementia (30.3%) entered only during the last 7 days of life, and 334 of 2429 (13.8%) only during the last 3 days of life. Model estimated mean Medicare hospice expenditures did not differ significantly by race or ethnicity: $4097 (95% CI, $3835-$4360) per dementia decedent among White beneficiaries, $3417 (95% CI, $2858-$3976) among Black beneficiaries, and $3372 (95% CI, $2605-$4140) among Hispanic beneficiaries (Figure 2).

### ED Visits and Hospitalizations by Race and Ethnicity

A higher proportion of Black and Hispanic decedents with dementia had ED admissions in the last 180 days of life compared with White decedents (645 of 809 [79.7%] and 274 of 357 [76.8%] vs 2753 of 3892 [70.3%]; *P* < .001) (Table 2). Even among dementia beneficiaries enrolled in hospice, 56 of 309 (18.1%) Black and 22 of 153 (14.4%) Hispanic decedents had ED visits at some point after hospice enrollment, compared with 191 of 1967 (9.7%) White decedents (*P* < .001). Admissions to the hospital after hospice enrollment were also more common among Black and Hispanic decedents than among White decedents (48 of 309 [15.5%] and 17 of 153 [11.1%] vs 119 of 1967 [6.0%]; *P* < .001). Overall, Black and Hispanic decedents with dementia had more ED visits (outpatient ED: 2.8 [2.2] and 2.4 [2.1] visits vs 2.5 [2.0] visits; *P* = .06; inpatient ED: 2.2 [1.4] and 1.9 [1.1] visits vs 1.7 [1.1] visits; *P* < .001), more hospitalizations (2.4 [1.5] and 2.3 [1.5] hospitalizations vs 2.0 [1.3] hospitalizations; *P* < .001), and longer hospital length of stay (10.0 [8.5] and 9.6 [6.6] days vs 8.0 [6.6] days; *P* < .001) compared with White decedents. End-of-life outpatient ED expenditures were similar across ethnic and racial groups, whereas estimated mean inpatient expenditures were substantially higher among Black and Hispanic than among White decedents (Black, $23 279; 95% CI, $20 690-$25 868 per dementia decedent; Hispanic, $23 471; 95% CI, $19 532-$27 410 per dementia decedent vs White, $14 609; 95% CI, $13 800-$15 418 per dementia decedent) (Figure 2).

Dementia beneficiaries were most frequently hospitalized for circulatory system diseases at the end of life (1242 of 5270 [23.6%] hospitalizations among White beneficiaries; 330 of 1541 [21.4%] hospitalizations among Black beneficiaries; 117 of 650 [18.0%] hospitalizations among Hispanic beneficiaries), followed by respiratory (986 of 5270 [18.7%] hospitalizations among White beneficiaries; 223 of 1541 [14.5%] hospitalizations among Black beneficiaries; 128 of 650 [19.7%] hospitalizations among Hispanic beneficiaries) and infectious and parasitic diseases (598 of 5270 [11.3%] hospitalizations among White beneficiaries; 250 of 1541 [16.2%] hospitalizations among Black beneficiaries; 98 of 650 [15.1%] hospitalizations among Hispanic beneficiaries) (Table 3). Injury and poisoning, as well as genitourinary, digestive, endocrine, and neoplasm conditions were also common causes of end-of-life hospitalizations in this population. A sizable proportion of these hospitalizations were for potentially avoidable conditions (eTable 2 in the Supplement).

**Table 3.** Major Causes of Hospitalizations in the Last 180 Days of Life Among Beneficiaries With Dementia by Race and Ethnicity

Disease category^a^	Hospitalizations, No. (%)			
	Non-Hispanic White (n = 5270)	Non-Hispanic Black (n = 1541)	Hispanic (n = 650)	*P* value
Circulatory	1242 (23.6)	330 (21.4)	117 (18.0)	.003
Respiratory	986 (18.7)	223 (14.5)	128 (19.7)	<.001
Infectious and parasitic	598 (11.4)	250 (16.2)	98 (15.1)	<.001
Injury and poisoning	495 (9.4)	97 (6.3)	51 (7.9)	<.001
Genitourinary	407 (7.7)	158 (10.3)	59 (9.1)	.005
Digestive	370 (7.0)	85 (5.5)	42 (6.5)	.11
Endocrine	215 (4.1)	96 (6.2)	36 (5.5)	.001
Neoplasms	221 (4.2)	82 (5.3)	27 (4.2)	.16

^a^ The table is restricted to major causes of hospitalizations accounting for ≥5% of admissions in any of the analyzed racial or ethnic groups. Principal discharge diagnoses were used to categorize causes of hospitalizations based on the Agency for Healthcare Research and Quality (AHRQ) Clinical Classifications Software (CCS) for *International Classification of Diseases, Ninth Revision, Clinical Modification *(*ICD-9-CM*) codes and the Clinical Classifications Software Refined (CCSR) for *ICD-10-CM* codes.

### End-of-Life Care Preferences by Race and Ethnicity

Treatment preferences for end-of-life care among dementia decedents differed substantially by race and ethnicity (Table 2). The proportion of dementia beneficiaries completing advance care planning was lower among Black (146 of 704 [20.7%]) and Hispanic beneficiaries (66 of 308 [21.4%]) compared with White beneficiaries (1871 of 3274 [57.1%]). A higher proportion of Black and Hispanic decedents with dementia had written instructions choosing all care possible to prolong life (30 of 144 [20.8%] and 12 of 65 [18.5%] vs 72 of 1852 [3.9%]), whereas a higher proportion of White beneficiaries preferred to limit care in certain situations (1708 of 1840 [92.8%] vs 114 of 141 [80.9%] and 51 of 64 [79.7%]), withhold treatments (1448 of 1799 [80.5%] vs 87 of 140 [62.1%] and 41 of 62 [66.1%]), and forgo extensive life-prolonging measures (1712 of 1838 [93.1%] vs 120 of 138 [87.0%] and 54 of 65 [83.1%]).

## Discussion

Our findings showed substantial ethnic and racial differences in dementia end-of-life care utilization patterns and patient treatment preferences. Black and Hispanic decedents with dementia used less hospice but more ED and inpatient services, and incurred roughly 60% higher Medicare inpatient expenditures at the end of life compared with White decedents. Among hospice users, more Black and Hispanic than White decedents with dementia were subsequently admitted to the ED or hospital before death.

The trends in racial and ethnic differences in end-of-life care observed in our sample of dementia beneficiaries are not entirely surprising given the health care utilization patterns in the overall Medicare population.^18,26,27^ Nonetheless, to our knowledge, this is the first study to quantify such differences among racially and ethnically diverse Medicare decedents with dementia in both community and nursing home settings. Understanding how and when individuals with dementia use hospice and other end-of-life care across populations is one of the highest research priorities identified by the National Academies of Sciences, Engineering, and Medicine.^28^ Our study provides new evidence highlighting unique end-of-life care utilization and treatment preferences across racial and ethnic groups among patients with dementia.

Race and ethnicity are known to be associated with end-of-life care type and intensity independent of other social determinants of health.^11,27,29,30,31^ Studies have suggested several possible reasons, in addition to cultural and religious or spiritual values. For instance, mistrust of the health care system due to medical racism and health inequalities in the US may lead more non-White patients to perceive hospice care as “giving up” and motivate them to request more aggressive, life-sustaining interventions.^18,32,33^ Our data showed that although most dementia beneficiaries desire less invasive care at the end of life, preferences to limit care, withhold treatment, or forgo life-prolonging measures were much less common among Black and Hispanic patients.

Additionally, such mistrust may compromise patient-physician communication, reducing access to high-quality end-of-life care. Researchers have documented racial and ethnic inequity in the provision of, and access to, hospice care.^34^ In our data, it is unclear which beneficiaries with dementia and family caregivers had physician counseling about end-of-life care, but we found that only 1 in 5 Black and Hispanic decedents with dementia completed advance care planning—a much lower proportion than their White peers.^30^ These written instructions give persons with dementia a voice in making end-of-life medical decisions when they become unable to understand or speak for themselves. Dementia education programs should foster community outreach and help patients with dementia and their caregivers understand end-of-life care issues, including the benefits of advance care planning.^30^

Hospice care may improve quality of life of terminally ill patients and their caregivers and reduce Medicare costs.^35,36,37,38,39^ In our sample, fewer than half of Medicare decedents with dementia used hospice services during the last 6 months of life. Although hospice is not always the right choice for terminal patients, more beneficiaries with dementia could appropriately benefit from the service. In particular, our results highlighted 2 concerning trends that may limit the full benefits of hospice received by dementia beneficiaries and caregivers. First, the timing of hospice enrollment was suboptimal. We found that almost 1 in 3 beneficiaries with dementia entered hospice very close to the end of life (ie, within 7 days before death) and for a short time period. This proportion was higher compared with the general Medicare population.^3^ Second, some dementia beneficiaries in hospice, especially Black and Hispanic beneficiaries, were subsequently admitted to the ED or hospital before death, suggesting a live discharge. Overall, we found that beneficiaries with dementia had similarly frequent ED visits and hospitalizations at the end of life compared with the general Medicare population.^6^ Health care professionals make decisions prospectively about whether to admit patients with dementia to the hospital. Some hospitalizations are necessary and expected as part of the natural course of treatment, whereas others may be considered low-value at the end of life. These transitions not only impose burdens on older adults with dementia and their caregivers but also increase mortality and costs.^6,40^ Medicare should consider alternative payment models, such as bundling services into an episode of care,^41^ as one way to address suboptimal hospice use and burdensome end-of-life transitions among dementia beneficiaries. These strategies would help incentivize high-quality services, reduce low-value, unnecessary ED visits and hospitalizations,^41^ and promote end-of-life discussions. Such discussions may reduce aggressive medical interventions near death and facilitate earlier hospice referrals.^39^ Medicare could provide more comprehensive reimbursement to hospital-based palliative care teams^6^ and reward hospitals for improved access to and delivery of effective hospice care for dementia patients.

### Limitations

This study had several limitations. First, we could not ascertain cause of death, a factor that may affect end-of-life care.^18^ We did not use diagnoses reported on death certificates because those records likely underreport dementia as an underlying cause of death.^42^ Future investigations would benefit from additional efforts, such as expert reviews of death certificates, proxy interviews, and medical record reviews, to determine how our findings differ by cause of death. Second, we relied on diagnosis codes in claims data to identify patients with dementia because HRS lacks a direct measure of dementia status and because there is no uniformly accepted case definition for dementia in observational studies. Our sample may have omitted individuals with undiagnosed dementia, of which a higher proportion are non-White individuals.^22^ Third, our sample had too few Asian Americans or patients from other racial or ethnic groups to allow further analyses. Fourth, our analyses were limited to fee-for-service beneficiaries aged 65 years or older, and thus the results might not fully generalize to Medicare Advantage enrollees or younger patients. Fifth, our data spanned from 2000 to 2016; we did not evaluate changes over time in hospital or doctor behavior or Medicare enrollment. For example, enrollment in Medicare Advantage has increased substantially over the last decade, especially among non-White beneficiaries.^43^ Finally, preferences for end-of-life care were based on proxy reports after the study participant’s death (ie, HRS did not collect this information prospectively)^25^ and therefore recall bias was possible.

## Conclusions

Although use of Medicare hospice benefits has increased,^3^ our results suggest that end-of-life care for beneficiaries with dementia remains suboptimal, especially for Black and Hispanic patients. The type and intensity of care individuals receive at the end of life is a complex issue, even more so among older adults with dementia because of their impaired ability to plan and communicate care preferences. Doctors and staff should address these communication barriers, especially in underresourced racial and ethnic communities. Medicare should consider alternative payment models to promote culturally competent end-of-life care and reduce low-value interventions and costs among the dementia population.

## Notes

Supplement. **eFigure.** CONSORT Diagram **eTable 1.** Odds Ratios of Hospice Use in the Last 180 Days of Life Among Medicare Decedents With Dementia **eTable 2.** Proportions of End-of-Life Hospitalizations for Potentially Avoidable Conditions
