# Supplementary material for: Racial and Ethnic Differences in Hospice Use and Hospitalizations at End-of-Life Among Medicare Beneficiaries With Dementia
Source: JAMA Netw Open. 2022 Jun 9;5(6):e2216260. doi: 10.1001/jamanetworkopen.2022.16260 (PMC9185179; doi:10.1001/jamanetworkopen.2022.16260)
Supplement: Supplement. — eFigure. CONSORT Diagram eTable 1. Odds Ratios of Hospice Use in the Last 180 Days of Life Among Medicare Decedents With Dementia eTable 2. Proportions of End-of-Life Hospitalizations for Potentially Avoidable Conditions [file jamanetwopen-e2216260-s001.pdf]

Supplemental Online Content

Lin PJ, Zhu Y, Olchanski N, et al. Racial and ethnic differences in hospice use and hospitalizations at end-of-life among Medicare beneficiaries with dementia. *JAMA Netw Open.* 2022;5(6):e2216260. doi:10.1001/jamanetworkopen.2022.16260

eFigure. CONSORT Diagram

eTable 1. Odds Ratios of Hospice Use in the Last 180 Days of Life Among Medicare Decedents With Dementia

eTable 2. Proportions of End-of-Life Hospitalizations for Potentially Avoidable Conditions

This supplemental material has been provided by the authors to give readers additional information about their work.

© 2022 Lin PJ et al. *JAMA Network Open.*

eFigure. CONSORT Diagram

1. Health and Retirement Study participants, 1992-2016 N = 42,719
2. With linked Medicare fee-for-service and/or Medicaid claims N = 26,044
3. Deceased in 2000-2016 N = 11,285
4. ≥6 months of continuous enrollment in Medicare prior to death N = 10,953
5. Aged 65 and older at death N = 10,564
6. Dementia diagnosis in claims[1] N = 5,164
7. With known race and ethnicity N = 5,058

[1]We identified dementia diagnoses using the International Classification of Diseases, Ninth Revision, Clinical Modification codes (331.0, 331.11, 331.19, 331.2, 331.7, 290.0, 290.10, 290.11, 290.12, 290.13, 290.20, 290.21, 290.3, 290.40, 290.41, 290.42, 290.43, 294.0, 294.10, 294.11, 294.20, 294.21, 294.8, 797, 331.82, 331.89, 331.9, 290.8, 290.9, and 294.9) and the Tenth Revision codes (G30.0, G30.1, G30.8, G30.9, F01.50, F01.51, F02.80, F02.81, F03.90, F03.91, F04, G13.8, F05, F06.1, F06.8, G31.1, G31.2, G31.01, G31.09, G94, R41.81, R54, G31.83, G31.89, and G31.9).

© 2022 Lin PJ et al. *JAMA Network Open.*

eTable 1. Odds Ratios of Hospice Use in the Last 180 Days of Life Among Medicare Decedents With Dementia

	**Odds ratio**	**95% CI**
**Race/Ethnicity**		
Non-Hispanic white (reference)	-	-
Non-Hispanic Black	0.65	0.55-0.78
Hispanic	0.84	0.66-1.08
**Age, years**		
65-74 (reference)	-	-
75-84	1.39	1.12-1.72
85+	1.39	1.13-1.71
**Female vs. Male**	1.19	1.05-1.35
**Education**		
Less than high school	-	-
High school	1.17	1.01-1.36
More than high school	1.32	1.13-1.54
**Cognitive score**[1]	1.51	1.02-2.23
**ADL limitations**	1.02	0.98-1.06
**IADL limitations**	1.07	1.01-1.12
**Comorbidities**	0.98	0.94-1.02
**Medicare-Medicaid dual eligibility**	0.87	0.74-1.01
**Nursing home resident**	0.81	0.71-0.93
**Proxy respondent**	0.84	0.71-0.99
**Constant**	0.42	0.29-0.61

ADL: activities of daily living, ranging from 0-6, with higher numbers indicating more limitations; IADL: instrumental activities of daily living, ranging from 0-5, with higher numbers indicating more limitations.

[1]Cognitive impairment was measured on a 0-1 scale by combining normalized Telephone Interview for Cognitive Status (TICS) scores and Informant Questionnaire on Cognitive Decline in the Elderly (IQCODE) scores, with higher scores representing more severe impairment.

© 2022 Lin PJ et al. *JAMA Network Open.*

eTable 2. Proportions of End-of-Life Hospitalizations for Potentially Avoidable Conditions

	**Non-Hispanic whites (n=5,270 hospitalizations)**	**Non-Hispanic Blacks (n=1,541 hospitalizations)**	**Hispanics (n=650 hospitalizations)**	**p-value**
**Circulatory**	**1,242 (23.6)**	**330 (21.4)**	**117 (18.0)**	**<0.01**
Hypertension[1] and heart failure[2]	441 (8.4)	122 (7.9)	52 (8.0)	0.83
**Respiratory**	**986 (18.7)**	**223 (14.5)**	**128 (19.7)**	**<0.01**
Asthma/COPD[3] and bacterial pneumonia[4]	212 (4.0)	37 (2.4)	24 (3.7)	0.01
**Genitourinary**	**407 (7.7)**	**158 (10.3)**	**59 (9.1)**	**<0.01**
Urinary tract infection[5]	219 (4.2)	81 (5.3)	22 (3.4)	0.08
**Endocrine**	**215 (4.1)**	**96 (6.2)**	**36 (5.5)**	**<0.01**
Diabetes[6]	47 (0.9)	42 (2.7)	13 (2.0)	<0.01

We measured hospitalizations for potentially avoidable conditions as defined by the AHRQ Prevention Quality Indicators (PQI) Version 2021:
https://www.qualityindicators.ahrq.gov/Modules/PQI_TechSpec_ICD10_v2021.aspx

[1]PQI 07: Hypertension
[2]PQI 08: Heart failure
[3]PQI 05: Asthma or chronic obstructive pulmonary disease
[4]PQI 11: Bacterial pneumonia
[5]PQI 12: Urinary tract infection
[6]PQI 01: Diabetes short-term complications; PQI 03: diabetes long-term complications; PQI 14: uncontrolled diabetes

© 2022 Lin PJ et al. *JAMA Network Open.*
